# A Fertile Ground for Ambiguities: Casual Sexual Relationships Among Portuguese Emerging Adults

**DOI:** 10.3389/fpsyg.2022.823102

**Published:** 2022-02-18

**Authors:** Rita Luz, Maria-João Alvarez, Cristina A. Godinho, Cicero R. Pereira

**Affiliations:** ^1^Faculdade de Psicologia, CICPSI, Universidade de Lisboa, Lisbon, Portugal; ^2^Católica Research Centre for Psychological, Family and Social Wellbeing, Universidade Católica Portuguesa, Lisbon, Portugal; ^3^Centro de Investigação e Intervenção Social, ISCTE, Instituto Universitário de Lisboa, Lisbon, Portugal; ^4^Instituto de Ciências Sociais, Universidade de Lisboa, Lisbon, Portugal; ^5^Departamento de Psicologia, Universidade Federal da Paraíba, João Pessoa, Brazil

**Keywords:** casual sex, casual sexual relationships, sexual behavior, emerging adults, college students, sociocultural context

## Abstract

Casual sexual relationships (CSRs) are frequent relationship experiences in young adulthood that provide opportunities for many to explore sexual relationships and to construct their sexual identity. Empirical research on casual sex is still lacking outside North-American countries, despite evidence pointing to the need to contextualize sexual interactions in their own sociocultural context. In order to better understand casual sexual relationships, these should be examined in with novel samples in other countries where a “hookup culture” as it is described in the North-American university campus is apparently absent. Through a qualitative study, we explored what casual sexual relationships consist of according to the perceptions of Portuguese college students (*N* = 35). The thematic analysis of eight focus group interviews resulted in the generation of six themes, three of which are presented here: (1) *What CSRs are*, regarding features and types of CSRs, (2) *Why individuals engage in CSRs*, focusing on positive and negative motivations, and (3) *What one gets from CSRs*, focusing on positive and negative outcomes of CSRs. Our findings showed that Portuguese emerging adults are familiarized with CSRs, particularly with *one-night stand*, *friends with benefits* and “*curte*”/*hookup*. Sexual interactions associated with other CSRs, such as *booty call* or *fuck buddies,* were mentioned but rarely associated with a distinctive label and established characteristics. Participants described the CSRs in a partially overlapping manner presenting some areas of ambiguity, such as with regard to sexual exclusivity and still-unlabeled sexual interactions. CSRs are generally evaluated as positively motivated and mainly beneficial. This study adds to the literature around casual sexual relationships by exploring and describing CSRs in a different sociocultural context, as well as indicating directions for future research in order to better prepare and empower young adults in their sexual and relational trajectories.

## Introduction

Over the past two decades, researchers have been investigating casual sexual relationships (CSRs), as these are increasingly frequent and openly discussed, especially among college students (e.g., Garcia et al., 2012). CSRs are understood as one-time or repeated sexual encounters between two individuals with varying levels of acquaintance, from strangers to friends, that may include a broad range of sexual practices, from kissing or embracing to intercourse, without emotional intimacy or expectations of romantic commitment (Wentland and Reissing, 2011; Alvarez et al., 2019).

Multiple theoretical frameworks have been used to explain involvement in CSRs by young adults. One of them is Arnett’s conceptualization of emerging adulthood as a period of time for exploration and experimentation in terms of sexual and relationship partners, and the construction of a sexual identity (Arnett, 2015). From this perspective, CSRs have an important function particularly from the late teens through at least the mid-20s, when individuals are focused on achieving a certain level of education and investing in their careers rather than seeking a romantic partner (Lyons et al., 2014, 2015). Enabled by social changes bringing the dissolution of sexual prohibitions and acceptance of new contexts for pursuing intimate and sexual relationships (e.g., Conley et al., 2013), CSRs are a way for a number of individuals to try out various types of relationships and forms of sexual involvement while maintaining the focus on their academic or professional goals (Hamilton and Armstrong, 2009). Additionally, for those who prefer casual over committed sexual relationships, CSRs proved to be a significant source of affection, providing the physiological and emotional benefits of intimacy associated with sexual activity (Garcia et al., 2018).

Researchers on romantic and sexual relationships have gone beyond a two-pole perspective on relationships to consider the existence of several possible relational arrangements positioned between committed relationships and one-night stands (Jonason et al., 2009). Four main types of CSRs have been reported in the literature: *one-night stand*, *friends with benefits*, *booty call*, and *fuck buddies* (e.g., Wentland and Reissing, 2011). The *one-night stand* is primarily defined as a one-time sexual relationship between strangers or brief acquaintances in a social setting (Singer et al., 2006). Being *friends with benefits* is characterized by planned, regular sexual activity between friends without romantic expectations or commitment (Afifi and Faulkner, 2000). *Booty call* is described as “a communication initiated toward a non-long-term relationship partner with the urgent intent, either stated or implied, of having sexual activity and/or intercourse” (Jonason et al., 2009, p. 462). Finally, *fuck buddies* have more regular encounters than *booty calls*, but despite the time spent together, partners invest more in the sexual relation than in establishing a bond of friendship (Wentland and Reissing, 2011). Findings also pointed to the existence of some degree of overlapping between these types of CSRs, which made it important to clearly acknowledge the differences and similarities between encounters (Claxton and van Dulmen, 2013; Rodrigue et al., 2015). This endeavor was undertaken firstly by Wentland and Reissing (2011), who described a group of dimensions (friendship, frequency of contact, type of contact, personal disclosure, and discussion of the relationship) along which the four types of CSRs could be distinguished. Later, through quantitative data, Rodrigue et al. (2015) identified a number of features (number of sexual partners in the past year, intention to have other sexual contacts with the partner, being under the influence of alcohol or drugs during the most recent sexual encounter, and type of sexual exclusivity agreement) that made it possible to identify five relationship profiles, two of them distinct from those previously cited as: the *ex-romantic partnership*, in which sexual encounters continue to take place after the end of the romantic relationship, and the *intimate and sexual partnership*, a hybrid of friendship and romantic relationships in which partners have frequent sexual and social interactions without intentions of becoming committed. These contributions provided a better comprehension of CSRs, clarifying their underlying motivations as well as similarities and distinctions between them.

According to social constructionism, individuals build their knowledge and make sense of the world through language by interpreting and classifying events and persons in categories (DeLamater and Hide, 1998). When this knowledge is shared, it becomes institutionalized and leads to habituation, making behaviors predictable and familiar activities more accessible. Following this paradigm, Simon and Gagnon (1986) suggested that sexuality is culturally conditioned that it depends on how certain behaviors and interactions are defined and labeled as sexual by individuals, with sexual behavior and expectations, like other social interactions, being guided by scripts (Simon and Gagnon, 1986; Gagnon 1990). Sexual scripts are mental representations that operate at cultural, interpersonal, and intrapersonal levels to guide expectations around sexual behavior and to anticipate the sequence of predictable behaviors in a cognitively efficient manner (Langer, 1978; Alvarez and Garcia-Marques, 2008; Olmstead et al., 2019). Through repeated experiences, cultural norms, or social learning, people develop scripts for sexual interactions and act accordingly.

Research on CSRs, conducted primarily in North-American countries, has identified a script for casual sex that encompasses specific behaviors, interactions, and contexts (Epstein et al., 2009; Olmstead et al., 2019) and is associated with a “hook-up culture” that has become socially established on university campuses as an expression of the normative and even compulsory nature of casual sex among university students (Bogle, 2008; Wade, 2017). Even though not all emerging adults engage in the campus-based hookup culture, such as those who do not attend college, those who are in a monogamous relationship, or those who choose to prioritize their studies and opt out, 60–80% of North-American college students report having been involved in at least one casual sexual relationship (Garcia et al., 2012; James-Hawkins, 2019). Although hookup scripts contain some distinctive components (e.g., Eaton and Rose, 2012; Olmstead et al., 2019), they are more varied and complex than dating scripts (Bogle, 2008; Heldman and Wade, 2010), making it more difficult to predict the pathways of the relationship (Allison, 2019).

The influence of the sociocultural environment on sexual scripts and on sexual behavior is well established, and there is evidence for cross-cultural differences in casual sex behaviors (e.g., Kaspar et al., 2016). Empirical research outside the North-American cultural context is still limited, however. In Portugal, where 33–52% of college students refer to having had a CSR (Reis et al., 2012; Alvarez et al., 2021a), there are few studies identifying and characterizing the different types of CSR, except for a few papers (Alvarez et al., 2021a). We apparently do not have a “hookup culture” as described in the North-American context, and engaging in CSRs in emerging adulthood is not perceived as normative worldwide. Moreover, most Portuguese college students (about 70%)^1^ continue to live with their parents, while most North-American students (72.5%)^2^ live on campus or off-campus away from their parents. The present study aims to fill this gap by examining different types of CSRs in a novel sociocultural context under the assumption that this needs to be considered when examining CSRs (Farvid and Braun, 2017).

As in many other Western societies, social and relational organization and sexual behavior in Portugal have undergone major changes in recent decades. In terms of sexual morality, a strictly conservative structure guided by the orientation of the Catholic Church has given way to a more open-minded society with less obvious differences in sexual standards for men and women, especially in the younger generations (Ferreira and Villaverde Cabral, 2010). Evidence of these changes is that although getting married and/or starting a family are still important projects for most young adults, they have been postponed, as shown by the significant increase in the average age at marriage and first childbirth. Younger generations seem to prioritize certain life tasks and also value the experience of different relationship alternatives before marriage (Aboim, 2005).

In previous work by our research team, we found that three types of CSRs—*friends with benefits*, *one-night stand*, and *making out*—are more salient for Portuguese emerging adults (Alvarez et al., 2019); we also concluded that each CSR was associated with a consensual label and definition, and understood as more different than similar according to a set of psychoemotional, behavioral, and sexual characteristics (Alvarez et al., 2021a).

With this study, we aimed to go a step further and provide a more thorough description of CSRs. Specifically, we aim to deepen knowledge about what CSRs consist of from the perspective of a group of Portuguese college students. We adopted a qualitative approach to elicit social actors’ narratives about casual sexual relationships. We were interested not only in the breadth of information, but also in the depth and insights that emerge from the discussion between participants. We therefore chose to conduct focus group interviews, which allowed us to address socially shared perceptions and opinions about CSRs, rather than idiosyncratic experiences. Focus groups enhance participants’ understanding of the topic through the group effect, the shared information that emerges only through group interaction (Noar et al., 2012), allowing us to learn about the most typical language, terms, or expressions that participants use in a context where they feel comfortable talking about sexual behavior (Frith, 2000). As participants’ contributions during group discussions were rich and varied, only a part of the findings will be presented in this article.

## Materials and Methods

### Participants

The study comprised 35 college students, aged between 18 and 28 years (*M* = 20.89, *SD* = 2.17), 19 of them women (see Table 1). All participants had sexual intercourse, with an average of 6.63 sexual partners (*SD* = 8.07, *Min* = 1, *Max* = 40), and most of the participants (26; 74.3%) had been involved in at least one CSR (*M* = 7.62, *SD* = 10.44, *Min* = 1, *Max* = 50). At the time of the focus group interview, three participants were involved in one CSR and one participant was involved in multiple CSRs.

**Table 1:** Demographic characteristics of the sample (*N* = 35).

	*M* ± *SD* (max, min)
Age of first intercourse	16.37 ± 1.4 (13, 19)
Age of first CSR	16.65 ± 2.02 (12, 20)		**N (%)**
Gender	±
Female	19 (54.3)
Male	16 (45.7)
Other	0
Ethnicity	
White	33 (94.3)
African-European	1 (2.9)
Latino/a	1 (2.9)
Religion	
Non-practicing	16 (45.7)
Catholic	17 (48.6)
Other	1 (2.9)
Not specified	1 (2.9)
College year	
Undergraduates	29 (85.3)
1st year	5 (14.7)
2nd year	12 (35.3)
3rd year	12 (35.3)
Post-graduates	5 (14.7)
Sexual Orientation	
Only same-gender/sex	2 (5.7)
Mainly same-gender/sex	0 (0)
Bisexual	2 (5.7)
Mainly other gender/sex	3 (8.6)
Only other gender/sex	28 (80)
Relationship status	
Single	10 (28.6)
In a relationship (dating)	22 (62.9)
Non-marital partnership	1 (2.9)
Did not answer	2 (5.7)

### Procedure

Following ethical approval by the [Faculdade de Psicologia da Universidade de Lisboa] review board, the authors contacted five higher education institutions in different regions of the country and received permission to recruit participants and collect data. The study was presented in each institution in prescheduled sessions with students during and after classes. In these sessions, it was made explicit that the aim of the study was to understand existing perceptions and opinions about CSRs through focus group interviews. Information was provided about criteria for participating, ethics, procedures for scheduling and attending focus groups, and reward for participation (a 10€ voucher). Inclusion criteria were being aged between 18 and 29 years, speaking European Portuguese as their native language, and having had at least one sexual experience. The exclusion criterion was living in the country for less than a year, as this was considered to imply an insufficient command of the language.

Multiple convenience sampling procedures were applied. Seventy-two students pre-enrolled, 36 were scheduled to focus group interviews according to their best convenience, and 35 participated in the focus group interviews. Concerning those who did not participate, the main reasons were not responding to the initial e-mails (*n* = 20), being unavailable on the proposed schedules (*n* = 8), and not completing the scheduling procedure (*n* = 4); one student could not participate because she was living in the country for less than a year, and another stated no longer being interested in participating. In the case of the online focus group, two participants were unable to guarantee privacy, and one scheduled participant arrived late and could no longer join the Zoom meeting to participate in the focus group interview. All participants signed an informed consent form before starting the group discussion.

The final sample consisted of participants from different regions of Portugal and was diversified in terms of gender, religiosity (practicing or non-practicing), relationship status, sexual orientation, and casual sex experience. Four same-gender (two female) and four mixed-gender focus groups were performed with 3–6 participants per group. Interviews had an average duration of 90 min (60–110 min) and took place during November and December 2019 in the higher education institutions where participants were recruited. Due to restrictions imposed by the COVID-19 pandemic, one focus group was performed in April 2020, using the Zoom platform. The groups were moderated by the first author, already experienced in conducting semi-structured interviews; in three groups, a post-graduation student assisted as co-moderator. Once the interview was finished, participants filled out a sociodemographic data questionnaire (see Table 1), and participants from the online focus group completed it using an online platform (Qualtrics).

### Instruments

All interviews were conducted using a semi-structured guide consisting of 15 questions prepared by the authors. The findings presented in this study derive from six of these questions (see Table 2). The interview guide was structured following guidelines described in the literature (Krueger and Casey, 2000). Questions to explore what constitutes CSRs were based on the literature reporting specific features of CSRs concerning initiation, code of conduct, communication, conflict management, sexual experience, and termination (e.g., Wentland and Reissing, 2011; James-Kangal and Whitton, 2019), and by the findings of previous studies conducted by our research team focused on the main temporal, psychoemotional, social, and sexual characteristics that help define and distinguish different types of CSRs (Alvarez et al., 2019; Alvarez et al., 2021a).

**Table 2:** Semi-structured interview guide.

Questions	Target information
1. What are casual sexual relationships and what characterizes these encounters?	Types of casual sexual relationships, names of the encounters, partners, duration, preferences, repetition of the encounter and/or of the partner, confidentiality, exclusivity agreement, contexts where CSR take place Dimensions used to characterize CSR and through which it can be distinguish different encounters
2. What makes someone want to have a CSR?	Reasons to have casual sex, what one seeks in CSRs, motivations, ideal outcome, no alternatives vs. preference for casual sex
2. What, how, and when does one communicate in CSRs?	Patterns and ways of communicating and what is shared in the context of CSRs
4. Can there be situations of disagreement or even conflict?	Causes of conflict in the context of CSRs and how these are handled
5. What kind of feelings and thoughts arise during encounters?	Positive and negative feelings and thoughts that may be present before, during, and after encounters; romantic interest after CSRs
6. What are the benefits and risks of CSRs?	Benefits and risks of engaging in casual sex

The facilitator began all interviews by welcoming participants, reminding them of the objectives of the study, and providing guidelines for the functioning of the group. To facilitate communication, all participants were first asked a neutral question about each one’s field of study, followed by a general question asking for a global definition of CSR. Participants were next asked key questions about the characteristics of CSRs without identifying a specific CSR, so that only the CSRs mentioned by the participants were examined. The moderator used probes, follow-ups, and unscripted questions to elicit further details and depth in a two-way dialogue (Krueger, 1998). Personal experiences were not requested, although participants were allowed to share them. Each interview was concluded with a brief summary of the main contributions, about which participants were invited to comment and share their thoughts and opinions.

### Data Analysis

Interviews were conducted in Portuguese, with audio recorded and transcribed verbatim. A thematic analysis of data was conducted, inspired by the guidelines proposed by Braun and Clarke (2006). To begin with, the first author read the interviews, using NVivo 12 software, identified, selected, and collected all excerpts containing participants’ accounts on CSRs. Afterward, through a deductive process, two different researchers per interview assigned codes to each excerpt. These codes were descriptors of the topics researchers intended to explore in the interviews, such as characteristics of CSRs, motivations for the involvement in CSRs, communication, disagreement and conflict, feelings and thoughts during an encounter, benefits and risks, and transitions between CSRs. Codes attributed by both researchers were then compared; considering the only moderate average level of agreement obtained in five interviews (*K* = 0.54), the disagreements were solved by a third researcher highly familiarized with the data and the initial codes; and in the other three interviews, the researchers met to discuss each case and find consensus.

After agreement was obtained, the first author grouped and repeatedly read and analyzed excerpts presenting the same code in order to identify the recurring ways people talked about CSRs; this author generated broader themes to structure the subjective understandings participants shared about CSRs. These themes were reviewed by the research team in a recursive and interactive process until reaching data saturation. Given the richness of data obtained and the specific goals of the present study, only the findings about what CSRs consist of and about reasons and outcomes of the involvement in CSRs are here reported.

## Results

Qualitative analysis of the focus group interviews led to the production of a final list of six themes and corresponding subthemes. The findings reported here focus on three of these themes: *What CSRs are*, which relates to the characterization of CSRs, *Why individuals engage in CSRs*, focusing the positive and negative motivations to engage in CSRs, and *What one gets from CSRs*, focusing on positive and negative outcomes of CSRs (see Table 3). The other three themes are mentioned in the footnote of Table 3, and two of them are presented elsewhere (Alvarez et al., 2021b).

**Table 3:** Themes and subthemes.

Themes	Subthemes	Second-order subthemes
What CSRs are	General characteristics	
	Types of CSRs	One-night stand
		Friends with benefits
		“Curte”/Hookup
		Sex-friends/fuck buddies
		Booty call
		Other (be with/be together, not-assumed relationship, experimental-type relationship)
	Distinctive features	Frequency of encounters
		Partners’ acquaintance and emotional involvement
		Secrecy
		Exclusivity
		Defining rules
Why individuals engage in CSRs	Positive drives	Freedom
		Will, physical attraction, and excitement
		Search for novelty
		Developing sexuality and relationships
	Negative drives	Seeking social validation
		To fill a void
		Coping with relationship failure
		Social pressure
What one gets from CSRs	Positive outcomes	Carefree sexual relationships
		Ego-boost
	Negative outcomes	Negative interactions with sexual partner
		Dashed expectations
		Negative feelings
		Consequences of unprotected sex
		Emergence of romantic feelings

*The other three themes generated by the thematic analysis of focus groups interviews were as: How CSRs happen (sexual scripts), The Single Sexual Standard, and The Sexual Double Standard.*

Participants are identified by a code indicating gender (M-man; W-woman), participant’s age, composition of the focus group (sg = same-gender; mg = mixed-gender), and order number of the focus group interview (1–8). Superscripts^a,b^, and^c^ were used to differentiate participants of same gender and age in the same focus group.

### What CSRs Are

While sharing their perspectives on CSRs, women and men participating in the group discussions provided a significant extent of information that was organized in three main clusters: general characteristics, types of CSRs, and distinctive features.

#### General Characteristics

When asked about their conception of CSRs, participants referred to a set of features to define CSRs: “*it happens when two individuals feel sexually attracted to each other*” (M19-mg1) and “*meet occasionally*” (W24-mg1), “*just to have sex*” (M23-mg3), “*without any commitment*” (W19^a^-sg5), and “*nor romantic feelings*” (W19-mg4). A CSR “*can last long time*” (W19^a^-mg7) or “*it can happen only once*” (M20-sg2). Furthermore, a participant pinpointed that “*while in a committed relationship, partners are engaged in giving each other pleasure, in a casual relationship each one just wants to get off and move on*” (M23-mg3).

Participants also mentioned contexts where it is common to get involved in CSRs, namely, student parties and nightclubs, where the consumption of alcohol and drugs is frequent. College life, mainly the first year, was also indicated as a context where CSRs normally occur, as students enter in the freshman year “*thinking that it is going to be as they see in American movies with parties all the time (…). At least, from what I saw in welcoming dinners, people hook up a lot*” (M20-mg1). Additionally, the freshman year, “*it’s the first year of total freedom*” (W19^b^-sg5) and “*It’s the year when people engage in more casual relationships*” (W20^c^-sg5). Mobile dating apps and everyday social life were also mentioned as possible, but less-likely contexts for CSRs.

#### Types of CSRs

Participants designated different types of CSRs, usually using the most popular North-American expressions, as they are given in entertainment media. The Portuguese terms that came up (e.g., “*comer*”: *screw* and *fuck*) were not CSR-specific, as participants used them interchangeably and mainly referring to sexual interactions. Additionally, casual sexual partners are often referred to as “friends,” and the nature of the relationship must sometimes be deduced from non-verbal clues: “*We always say ‘oh, we are friends’. And then we let our smile speak for itself*” (M24-sg2). As one participant mentioned, the Portuguese “*glossary has a long way to go!*” (W19^a^-sg5), which may explain one participant’s uncertainty as he stated that “*it’s weird to describe everything that can happen in three or four words* (…) *we can never know very well, because each case is unique, as each person is unique, and each relationship is unique*” (M20-mg1).

The most frequently mentioned labels referring to casual sexual relationships were as: *one-night stand* (“*caso de uma noite*”)*, friends with benefits* (“*amigos coloridos*”), and “*curte.*” Descriptions of *one-night stand* and *friends with benefits* were equivalent to those appearing in studies conducted in other cultural context, especially in the United States; in the case of “*curte,*” it is a well-established Portuguese expression associated to the first sexual experiences occurring during adolescence. In our previous studies, the term “*curte*” was used to identify a casual sexual relationship that included different sexual practices, from kissing to hugging and touching, but excluded sexual intercourse, seeming equivalent to *making out*. Accordingly, in this study, it still remains perceived as “*the basic thing, like tongue-kissing*” (M19-mg1) occurring mainly during adolescence as discussed by two participants:

W21-mg3: *when people are younger they can have casual relationships without having sexual intercourse, they just kiss each other now and then, and nothing more (…) and this is called “curte.”*

M23-mg3: *But that is when you are 14 or 15 years old*!

However, this young man also underlined that “*mentalities are changing and sex* [i.e., sexual intercourse] *is becoming a commonplace*” (M23-mg3). This idea was confirmed by a young woman in a different focus group who shared that “*previously I thought ‘curte’ was just kissing and hugging, but now I’m changing my opinion and I think it also includes sexual intercourse*” (W20-sg6). It hence seems that the understanding of this CSR is evolving and it is becoming analogous to *hookup* as a “*deliberately vague term*” (James-Hawkins, 2019, p. 63) applied to an unspecified sexual behavior, from kissing to penetrative sexual intercourse (Olmstead et al., 2018). Those using this term (“*curte*”/*hookup*) mention the involvement in a sexual relationship without specifying the sexual behaviors they were engaged in because “*there is no pre-established pattern for everyone, because for me ‘curte’ can be one thing and for another person it can be only a kiss*” (W23-sg6). The following discussion well illustrates the ambiguous meaning of “*curte*”/*hookup*:

M20-mg1: “*Curte” implies that it is not an obligation nor partners have any responsibilities with each other, but it is sufficiently regular for both to expect that, when they meet, they’re going to get sexually involved with each other. It is not an obligation, but it* [sexual interaction] *always happens (…) and they may have sexual intercourse that depends on what they want.*

W24-mg1: *I would say that it is a synonym of friends with benefits, at least I use it that way.*

M19-mg1: *I thought it was a one-night stand, but I think that are just different perspectives on what happens…*

Other English expressions were used to identify CSRs, but they were less known by the participants. *Sex friends* were mentioned as equivalent to *friends with benefits—*“*I think it’s the same, friends with benefits and sex friends, basically they are friends but…* [also have a sexual relationship]” (M28-sg8) and *fuck buddies* were explained as resembling *friends with benefits* but revolving around sex: “*only the …* [sexual relationship]*, without the friendship*” (M20-sg2). *Booty call* was also presented and described by one participant as *“when you call someone* [to meet and have sex] *and that person may or may not want to*” (M28-sg8) and by others as “*in Tinder, people do not even have to talk, they arrange to meet up right away”* (M20-mg3) *That’s a booty call*” (W21-mg3). The typical interactions of a *booty call* were also described by another participant—“*they just have to send a text and if both want to, they meet and…*” (W20^a^-sg5)—but without a specific label associated.

In very particular cases, none of the previously Portuguese or English expressions suited, and new ones were mentioned. For instance, *be with/together* (“*estar com/junto*”) referred to a CSR where partners have social interaction and share interests other than sex-focused ones. In *not-assumed relationships* (“*relação não assumida*”), partners have romantic feelings for each other but prefer to keep the relationship informal/uncommitted and secret. An unlabeled experimental-type relationship was also mentioned, described as a short-term antecedent to a possible committed relationship during which partners get to know each other further and make decisions about becoming committed.

#### Distinctive Features

Participants described the three most mentioned types of CSRs according to their frequency, acquaintance among partners, emotional involvement, secrecy of the relationship, and exclusivity, which made it possible to obtain a thorough description of each one and, consequently, to distinguish them (see Table 4).

**Table 4:** Distinctive features and transitions between CSRs.

Features		Frequency of encounters	Partners’ acquaintance				Emotional involvement	Secrecy	Exclusivity	Defining rules	One-time	Recurrent	Strangers	Acquaintance	Friends	Couple-like	Yes	No	Sometimes	Yes	No	Yes	No	Sometimes	Yes	No	Sometimes	
CSRs	ONS	X		x					x			x		x			x	
	FWB		x			x		x			x				x	x		
	Hookup		x		x					x		x			x			x
	SF/FB		x															
	BC		x															
	Other		x				x	x			x		x			x		

*ONS, One-night stand; FWB, Friends with benefits; SF/FB, Sex-friends/fuck buddies; BC, Booty call; and Other, Be with/be together, not-assumed relationship, and experimental-type relationship*.

##### Frequency of Encounters

Whether sexual encounters occur only once or are recurrent over time is what differentiates the *one-night stand*, a spontaneous one-time encounter, from *friends with benefits* and “*curte*”/*hookup*, where the encounters are normally intentional and follow a repetitive pattern.

##### Partners’ Acquaintance and Emotional Involvement

Participants also considered that the type of CSR varies depending on the acquaintance and emotional involvement among partners as “*the type of [casual] relationship you have with someone you meet occasionally at parties is different from the relationship you have with someone who is in your friends’ group or studies in our college*” (M19-mg1). While a *one-night stand* may occur between strangers or people who do not know each other well, partners in “*curte*”/*hookup* are mainly acquaintances who hang out together at night and have little emotional involvement. Being *friends with benefits* was described as happening between friends who share deeper feelings for each other and have a more intimate relationship that for some represents an emotional safe haven: “*when we feel that something is missing, some lack* [of affection], *we go to see our little friend*” (W20^a^-mg4).

##### Secrecy of the Relationship

Regarding secrecy, while *one-night stands* and “*curte*”/*hookup* are usually acknowledged among others, even if details of the relationship may be kept unsaid, *friends with benefits* was considered secret in order to preserve the status quo within the circle of friends.

##### Exclusivity

Participants considered that there is no *a priori* exclusivity in CSRs and that a sexual agreement is normally absent or only implicit. However, some participants mentioned that it is plausible that individuals “*do not like to have two sexual partners simultaneously*” (M23-sg2) and prefer sexual exclusivity, mainly in long-term CSRs, as in *friends with benefits* or repeated *hookup* encounters, either as an assurance concerning health (preventing STIs) or psychosocial issues (being neither single nor unavailable), or as a confirmation that partners “*keep each other satisfied*” (W20^c^-sg5). In these cases, for a partner to engage in extra-dyadic casual sex is unexpected and of consequence, representing an adverse outcome and constituting a reason to end the relationship.

##### Defining Rules

As for sexual exclusivity, talking about the status and other rules of the relationship was considered vital in CSRs where encounters are repeated, as in “*curte*”/*hookup* and *friends with benefits*. Even if it may be perceived as “*taking the relationship too seriously*” (M20-mg1), participants highlighted that “*trust and communication are the keys*” (W20^a^-sg5) and “*people in this kind of relationship* [friends with benefits] *must define, in the beginning, the rules they will follow, and the communication must be clear and open*. *Otherwise it will not work*” (M22-sg8). Despite personal experiences not being explored, and the frequency and effectiveness of this type of communication remaining unclear, these personal accounts provide a glimpse of different approaches to this matter:

M23-mg3: (…) *in your case with your friend, you’re hanging out and maybe you’re thinking that you’re going to end up the night together, but maybe the guy is thinking that the night is almost over and he’s going to hookup with someone else, and you’re left twiddling your thumbs* [laughter]

M20-mg3: *If there are any expectations*…

W21-mg3: *That doesn’t work for me. I am really straightforward and if I go out to hang out with a friend with whom I am having sex, we say ‘Ok, tonight something is going to happen between us’… or not, or if we can be with other people or not. And for me, for both of us to always be on the same page and to know what is going to happen, so that none of us will end up twiddling our thumbs* [laughter]

Moderator: *So, what M23-mg3 was describing may happen if you don’t have this kind of communication, that’s it?*

M23-mg3: *That will really depend on people.*

W23-mg3: *But that way it seems that there is some kind of obligation…*

### Why Individuals Engage in CSRs

Young women and men in all focus groups agreed that engaging in CSRs is driven mainly by positive emotions and depends on each individual’s desires and goals. Nevertheless, with further discussion, some less-positive views about what makes a person want to get involved in a CSR also came up and were explored.

#### Positive Drives

##### Freedom

One participant explained that “*I am free, I want to be with someone I feel attracted to and that makes me feel good in that moment*” (M20-mg3), synthesizing what engaging in CSRs is all about for this group of college students. Accordingly, all participants considered that the involvement in CSRs was more dependent of personal choices rather than on social norms or any type of external pressure.

##### Will, Physical Attraction, and Excitement

A specific mindset was proposed for the involvement in CSRs: “*there must always be attraction and there must always be willingness*” (M19-mg1). Participants considered that CSRs are often driven by physical attraction and excitement: the pursuit of the other and the expectation of holding someone’s gaze, the playfulness of flirting, and the thrill of seducing and being seduced were viewed as a source of pleasure and joy. These were viewed as “*the first game, the most exciting part of it*” (M18-sg2). The assumption that CSRs are a journey of pleasure, wellbeing, and delight was a core motivation for individuals.

##### Search for Novelty

Participants considered casual sex to be motivated by the search for novelty as it provides opportunities to “*break from the routine*” (M20-mg7), “*meet new people,*” (W20^b^-sg5) and “*fool around*” (M23-mg3).

##### Developing Sexuality and Relationships

Some participants also mentioned that CSRs allow a process of field-based learning in sexuality. For some, *“friends with benefits relationships can be used to explore our sexuality. (…) That person can teach me to do this, I can teach that person to do this… I think it’s an exchange of knowledge, of experiences*” (W20^a^-sg5). For others, the fact that partners may be strangers makes it that “*casual sex can be convenient for someone who wants to try a new sexual practice but does not have the courage to do it inside a committed relationship*” (M22-sg8). CSRs were also viewed as a way to prepare for a romantic relationship as “*it’s important for people to know what type of person they like, what type of relationship they like, and this [casual] kind of relationships helps a person a lot to know and to understand. No one wants to get stuck in something they do not like*” (M28-sg8).

#### Negative Drives

##### Seeking Social Validation

While there generally were not major concerns about CSRs, after further discussion participants attributed a negative valence to some motivations for engaging in casual sex. It was considered that, as casual sex may function as an ego booster for some individuals, those with low self-esteem may enter into CSRs just seeking external/social validation.

##### To Fill a Void

Participants also suggested that the involvement in CSRs may arise from loneliness and lack of affection when the need to fill an emotional void motivates individuals to “*get involved with others even if they are not attracted, just out of despair*” (M19-mg1) in the case of a *one-night stand,* or in *friends with benefits*, “*when something is missing, we go to see our little friend*” (W20^a^-mg4). Aiming to initiate a committed relationship was also mentioned as a negative motive to engage in casual sex, as some individuals “*start to send messages and act very possessively (…) and these kind of things must be natural; you cannot force anything on anyone. That is very annoying*” (M20-mg1).

##### Coping With Relationship Failure

Casual sex was also portrayed as “*rebound sex, when people have a break-up and want to have fun*” (W23-mg3) or even as a strategy of emotional avoidance, when “*I could not trust anyone the same way. So, there yes, I had casual relationships. But casual sex for me meant that I would leave as soon as it was over, no hugs, nothing, because I did not want to… I was clearly protecting myself*” (M24-sg2).

##### Social Pressure

Most participants agreed that individuals are and feel free to decide whether they want to engage in casual sex or not. Nevertheless, some situations where social pressure may influence this decision were described as: some individuals may feel the urge to behave like others do because they “*see others having so much fun in these kinds of relationships*” (M20-mg7), or they may act because they feel “*some kind of pressure from people outside – I mean, I feel that, at our age, if we are not sexually involved with someone we are seen as losers*” (W21-mg3).

### What One Gets From CSRs

The involvement in CSRs was perceived as beneficial as the outcomes presented were mainly positive. In parallel, participants described a number of possible issues and negative consequences that may arise during and in the aftermath of a CSR. Nevertheless, while smaller in number, positive outcomes seemed more prevalent in most discourses. The following dialogue between two young women illustrates well the probationary and positive perspective of most participants on engaging in CSRs:

W23-sg6: *An experience is always an experience. If nobody gets hurt, or is called into question or disrespected, I think it is never a mistake, I think we always have something to learn from the situation. Because if we think about it, we may always make mistakes in relationships, casual or not, we will always make mistakes in some moments in our lives.*

W21-sg6: *We have to take the good out of it and see what we can learn from it.*

#### Positive Outcomes

##### Carefree Sexual Relationships

In CSRs “*We have the benefits of a committed relationship, without all its complications*” (W25-mg4)—this was a major outcome for most participants. In fact, contrary to committed relationships, which were described as constraining and time-consuming, CSRs represented “*an extra in our lives, that helps us to go through every day in a carefree way. We do not have to go deeper in terms of emotions or feelings… as long as we have the sexual part*” (M24-sg2).

##### Ego-Boost

Engaging in casual sex also seemed to constitute an empowering and ego-boosting experience. Participants of both genders considered that through the involvement in CSRs individuals validate their capability to seduce a potential partner: “*When he looks at you, you grow, you feel beautiful, no one can stop you!*” (W19-mg4) and “*it boosts your ego, to know that someone else has a sexual desire for you*” (M23-sg8).

#### Negative Outcomes

##### Negative Interactions With Sexual Partner

Although CSRs are characterized by some level of emotional detachment, the interactions with the casual sex partner seemed to influence the experiences of CSRs. A woman considered that poor treatment from a casual sex partner leads to negative feelings as *“it’s not pleasant if you get there, he/she does not even say hello or give you a glass of water, it’s just ‘Let us go to the bedroom’ and that’s all*” (W19^a^-mg7). A young man focused on the consequences of the lack of intimacy between casual sex partners and how it makes him feel the urge to “*immediately leave the room after sex, otherwise it becomes awkward*” (M23-sg2). Perhaps, the discontentment with relationship disengagement occurs because “*it’s rarer that people search only for sex. Because, for most of us, both parts come together, the affective and the sexual relations complement each other*” (W19^b^-mg7).

##### Dashed Expectations

Participants also stated that CSRs not always correspond to one’s expectations. Disappointment was associated with encounters “*that did not correspond to expectations, not emotionally, but mostly regarding the physical part*” (W23-sg6) or when “*the other person does not respond [to messages] … if this is not what you were expecting, (…) it can hurt more than being in a relationship and break up*” (W19^a^-sg5). There may also be some emotional turmoil when one person frequently engages in CSRs aiming to learn more about sexuality and relationships, and this ends up being a “*a double-edged sword as you may have many relationships thinking that you will know all of it for when you are in a committed one; but then things can happen otherwise, and you may find out that finally you do not want to get stuck with the same person”* (M24-sg2).

##### Negative Feelings

Participants pointed other negative feelings that may arise in the aftermath of the encounter “*when it starts weighing on our consciences*” (W20-sg6). Regret, disgust, or incredulity were mentioned regarding the chosen sexual partner: “*Sometimes, the day after, we think “What the fuck, how I was with that person?!*” (M19-mg1), mainly because many individuals are under the effect of drugs or alcohol when starting an encounter, leading to the point “*when you wake up, at home, and you do not remember anything from last night. Then someone texts you asking ‘What about it, bro, how did that go with that girl?’ and you think ‘What girl?!’* [laughter] *There, your world falls apart*” (M18-sg2).

##### Consequences of Unprotected Sex

Even if individuals seem to be well informed about potential negative health outcomes, “*there are people who think that nothing will happen to them and always have unsafe sex*” (M20-mg1). This underestimation of risks is more frequent when sexual partners are friends or have regular encounters as participants considered that “*if we avoid [sexual] relations with people we do not know…Always being with the same person reduces the risk*” (M23-sg8). Riskier behaviors were also attributed to the effects of alcohol or drugs—they may have trouble reasoning about the potential consequences of their actions, not taking the necessary precautions—and to the selection of hormonal or emergency contraception over condoms with the intent of avoiding an unwanted pregnancy but neglecting the risk of transmitting or getting a STI.

##### Emergence of Romantic Feelings

An issue considered important in CSRs was that “*many people really need love and affection and cannot keep the relationship casual because they start to have feelings*” (W21-mg3). It was stated that romantic feelings for one’s sexual partner may emerge during a *one-night stand*, as “*some people feel immediately attached*” (M19-mg1), mainly “*people who probably need to have something serious with someone, to find support*” (M20-mg1). However, participants considered that “*in friends with benefits it is more dangerous because it’s where people get to know each other better. In the other* [relationships] *sex is more a matter of physical attraction*” (M20-mg1). The major risk in *friends with benefits* is that the emergence of non-reciprocal romantic feelings may jeopardize the friendship, leaving both partners hurt, and it can even break ties inside the group of friends.

## Discussion

An in-depth understanding of CSRs implies knowing what they consist of according to the culturally determined perceptions of casual sex. With this study, we sought to achieve a deeper knowledge of CSRs based on the narratives and accounts shared by a group of Portuguese emerging adults. This goal was achieved as qualitative findings put in evidence the different types of CSRs and their main characteristics, as well as what are individuals looking for when they engage in CSRs and what they get from them, helping us to have a more comprehensive perspective on these relationships.

### Characterization of CSRs

Participants showed no difficulty explaining what CSRs are, using general features to describe CSRs that largely coincide with those presented in previous research (e.g., Claxton and van Dulmen, 2013; Wentland and Reissing, 2014): uncommitted relationships, mainly focused on spontaneous or sought-out sexual interactions, between two individuals who may know each other or not, most frequently in party and nightclub contexts, where alcohol and other drugs are consumed and play an important role.

During group discussions, three main types of CSRs were distinguished and labeled as: *one-night stand*, *friends with benefits,* and “*curte*”/*hookup*. *The one-night stand* is the most recognized CSR, probably because its features are the most prototypical of casual sex. *Friends with benefits* is also currently used by Portuguese emerging adults to label an ongoing CSR between two individuals who have a previous friendship, characterized by a deeper emotional bond and intimacy. Concerning “*curte*”/*hookup*, both the definition and the associated interactions remain adjustable to different sexual interactions, conferring a highly ambiguous meaning to this encounter. The clearest feature of this CSR is that sexual activities range predominantly among kissing, touching, and hugging, which is in line with previous findings, but in some cases, depending on the preferences of both partners, sexual intercourse may happen. While in our previous studies, “*curte*” was considered equivalent to *making out*, our present findings reflect a wider range of sexual behaviors that may occur in the context of this relationship and, consequently, that the understanding of it is evolving, which also implies an adjustment of its label and definition. It hence seems that “*hookup*” is the better equivalent English expression to “*curte*,” even if in some cases, “*curte*” was perceived as a *one-night stand* or as a *friends with benefits* relationship.

The ambiguity around certain labels, resulting from the variability of relational configurations concerning the number of encounters, sexual practices, or quality of relationship between partners (Holman and Sillars, 2012) is hence reinforced by unclear boundaries and subjective conceptualizations of some CSRs. This ambiguity is also patent in the occurrence of sexual situations corresponding to two of the main CSRs reported in the literature, *booty call* and *fuck buddies* (Jonason et al., 2009; Wentland and Reissing, 2014), that are only occasionally identified using a specific label. The unfamiliarity with these types of CSRs is not reported in the literature and may be attributed to limited cultural knowledge of the terms for these CSRs and to still-unfolding norms and meanings, lacking a label in Portuguese to identify them. Looking at the glass half full, the fact that less-known CSRs are being increasingly identified and described may be a sign of the development of an important body of social knowledge about casual sexual relationships, probably mostly influenced by what is shared in social networks and mass media (e.g., films and lyrics of popular songs), a “*popular culture representing aspects of actual contemporary sexual behavior and providing sexual scripts for emerging adults*” (Garcia et al., 2012, p. 161). Meanwhile, the existence of gray areas in the way emerging adults understand (or fail to understand) CSRs is a potential pitfall, as it may lead to individuals feeling insecure about or not recognizing the type of relationship they are involved in, its typical interactions and expected outcomes, which may lead to frustrated expectations and negative consequences in terms of psychological and sexual health.

Regarding (sexual) exclusivity and defining the rules of the relationship, the thoughts shared by our participants offer a perspective on CSRs less frequently found in the literature, one that reflects the complex and ambiguous character of CSRs. Such qualities are functional in making the involvement more acceptable and leaving the evolution of the relationship more open (Bogle, 2008; Kalish and Kimmel, 2011). Despite the validation of the dominant premise that casual sex implies neither commitment nor exclusivity, expectation of an exclusive sexual relationship exists and is perceived as rightful, either because of a personal preference, for health/psychosocial reasons, or as a logical consequence of both casual partners having their physical and psychosocial needs satisfied. Consequently, while absent in the *one-night stand,* agreement on sexual exclusivity in long-term CSRs like *friends with benefits* and *“curte”/hookup* is considered important so that both partners are aware of the rules and may act accordingly. Depending on the type of CSR, communication about the status and the rules of the relationship is also valued as a sign of trust and honesty about the nature of the relationship, as well as a way to manage friendship and sexual involvement in *friends with benefits* relationships.

Communication about sexual exclusivity and other rules in CSRs is important for the prevention of sexually transmitted infections (Mitchell et al., 2012) and also has an impact on individuals’ experience of CSRs (Knight, 2014). This is of foremost importance when considering the role attributed to these relationships by emerging adults in the development of sexual identity and readiness for future committed relationships. However, despite the relevance attributed to overt relational communication, the literature reports a lack of it in CSRs (James-Kangal and Whitton, 2019; Machia et al., 2020) and even the perceived incongruity between the effort involved in relational communication and the effortless nature of a casual sexual relationship itself (Knight, 2014). Even though participants consider the discussion of the status and rules of the relationship to be important in CSRs, our findings do not make it possible to clearly ascertain whether this concern translates into effective communication. If not, this may amplify the tension between expectations and reality, leading to disappointing outcomes, such as a poor sexual experience, health issues, or the abrupt termination of the relationship, with the psychological distress this may entail (Owen et al., 2013).

### Understandings About What Engaging in CSRs Is All About

Despite our main question being focused on what CSRs consist of, interrogation of how they are perceived in terms of the underlying motivations and potential outcomes helped us to shed a light on the role of this type of relationships for emerging adults in this sociocultural context.

According to the literature, sexual motivations are important determinants of wellbeing (Gravel et al., 2020), and individuals involved in CSRs may report both self-determined and non-self-determined motives (Deci and Ryan, 2000), oriented toward the pursuit of positive experiences and the avoidance of negative ones (Cooper et al., 1998). Hence, it seems positive that, in contrast to the North-American hook-up culture where “it feels wrong not to go along” (Wade, 2017, p. 87), for this group of Portuguese college students, besides being an end in itself, engaging in casual sex represents a way to socialize, relax, and have a good time. Even if first-year college students, based on what they see in entertainment media, can be more prone and even expecting to engage in casual sex in specific contexts (e.g., student parties), there does not seem to be a cultural norm that encourages or compels individuals to this type of sexual behavior. Accordingly, the majority of the declared motivations is self-determined and oriented toward the pursuit of positive experiences with physical pleasure and the quest for positive emotions as the most important drives for engaging in casual sex, as found in other studies (e.g., Armstrong and Reissing, 2015).

Engaging in CSRs is also viewed as a way to explore and learn more about sexuality and relationships. So, in contrast with the idea that involvement in CSRs may potentially interfere with developing the skills necessary for successful transitions into committed relationships (Claxton and van Dulmen, 2013; James-Kangal and Whitton, 2019), casual sex is perceived as an adequate strategy for young adults to learn and prepare themselves for romantic relationships, which is more in line with the exploration of sexual identity that occurs during emerging adulthood (Arnett, 2015).

Associated with these positive drives to engage in casual sex is the perception of it being mainly beneficial as it allows one to have sex while keeping one’s own freedom as well as it reinforces one’s sense of personal worth and self-confidence. However, the involvement in CSRs is not without risks, as it may also be driven by the need to either forget past negative relationships or avoid new ones, functioning as a strategy to cope with negative emotions, loneliness, and low self-esteem, or driven by extrinsic social goals, either appetitive (initiating a committed relationship) or aversive (social pressure to participate). In fact, negative outcomes were also mentioned, namely, concerning physical, sexual, and psychological health and wellbeing, which is also found in the literature (Bersamin et al., 2014; Wesche et al., 2020). The knowledge of what the involvement in CSRs implies, namely, the drives and the outcomes perceived either as positive or negative, may help us to better target sexual health and education interventions for individuals engaging in casual sex, especially for those feeling negatively affected by these types of experiences.

In sum, exploring the knowledge, perceptions, and opinions Portuguese emerging adults have about CSRs gives us a richer understanding of these relationships in their sociocultural context. By adopting a knowledge-building style, participants discussed and even argued over some topics, trying to organize and make sense of different perspectives. Portuguese young women and men are familiarized with CSRs and their characteristics as well as with the motives and outcomes associated with engaging in casual sex, which have similarities with those reported in studies conducted in other cultural settings. In contrast, narratives about CSRs suggest the absence of strict norms, accompanied by vague boundaries between relationships and, in some cases, still-unlabeled casual sexual interactions. Young women and men must hence deal with some level of ambiguity when navigating casual sexual relationships, following subjective rules about how to behave and what to expect from the sexual situations in which they are involved. Despite the underlying risks, it is possible to anticipate from this scenario, such as unmatched expectations, participants shared an overall positive attitude toward engaging in casual sex. Unlike what is described for the North-American college campuses, Portuguese college students do not recognize a ubiquitous cultural norm making the involvement in CSRs felt as compulsory, which lead us not to identify a hookup culture. In fact, CSRs are perceived as mainly self-determined and beneficial for individuals’ wellbeing and self-esteem, and as a field-learning process in sexuality and relationships.

## Limitations and Future Directions

The study presents some limitations that must be considered. First, although quotas for participants’ gender and age were outlined, the sample was self-selected. It must hence be considered that the overall positive perceptions and attitudes toward CSRs found in the current study are not free from a volunteer bias as participants in sexuality studies tend to hold attitudes toward sexuality and sociosexuality that may amplify the perspective found (Wiederman, 1999). Second, although an effort was made to promote open exchanges between participants, the group context may have influenced participants in the selection of information they shared, either by not having the opportunity, not feeling comfortable, or because of the pressure to conform with others’ opinions. In the same vein, social desirability may have guided the contributions of some participants, as the interviewer’s gender (woman) may have acted as a bias factor, leading participants to respond according to their perception of the interviewer’s expectations. Additionally, in some groups, participants were fellow students or acquaintances, which may have lead them, through identification, to share opinions in agreement with others (Kelman, 1958). Third, although it was not a goal of this study to obtain generalizable results, the sample was rather homogenous in terms of ethnicity, educational level, and occupation. Concerning the latter, all participants were college students, which also implies that findings may reflect the perception and opinions inside this specific social group rather that of emerging adults overall. However, the few existing studies comparing college students and non-students find more similarities than differences (Olmstead et al., 2019). Nonetheless, future studies should include participants from more diversified sociodemographic backgrounds to allow a wider exploration of perceptions and meanings of casual sex.

Other methodological considerations must be made. Intending to create different relational contexts where a maximum of diversified and even contrasting perspectives on CSRs might arise, we opted to conduct same- and mixed-gender focus group interviews. Considering the objective of the present study, we did not examine the gendered knowledge and perception of CSR. However, a gender analysis of the data was conducted and is presented elsewhere (Alvarez et al., 2021b).

Additionally, assuming the risk that some encounters could remain unaddressed, as the terms and vocabulary to describe them may be insufficient, we chose to explore only the CSRs mentioned by participants. Similarly, other topics that would be important for the understanding of CSRs but that were unknown or considered irrelevant by participants were not further explored, in the interest of obtaining the most accurate representations of CSRs from the participants’ perspectives, rather than directing their discourses. In future studies, these aspects as well as other CSRs should be included, and sexual behavior should be further investigated, given that it was not explored by our participants despite its centrality in casual sex, using individual interviews in order to obtain other kinds of detailed and reliable contributions.

## Conclusion

This study contributes to a deeper understanding of casual sexual relationships in a different sociocultural context than those previously reported in the literature. This provides a more nuanced understanding of CSRs in a specific non-North-American cultural context where most college students continue to live in their family home. Our findings highlight that, although a set of typical characteristics can be attributed to CSRs, they are characterized by vague boundaries and subjective norms, which entails a meaningful level of ambiguity regarding encounters and consequent tension between expectations and outcomes of casual sex. The perception of CSRs as positive and significant experiences supports the idea that these relationships are of major importance during emerging adulthood as they contribute to the construction of sexual identity. Practical reflections may be drawn from the current study, especially concerning effective communication among partners in order to clarify the sexual situation in which they are involved, with a view to preventing unwanted experiences and promoting healthy and safer sexual relationships.

## Data Availability Statement

The raw data supporting the conclusions of this article will be made available by the authors, without undue reservation.

## Ethics Statement

The studies involving human participants were reviewed and approved by Comissão de Ética e Deontologia do Conselho Científico da Faculdade de Psicologia da Universidade de Lisboa. The patients/participants provided their written informed consent to participate in this study.

## Author Contributions

M-JA, CP, CG, and RL worked on the conception and design of the study. RL and M-JA proceeded with recruitment of participants and conceived the instruments for data collection (semi-structured interview guide). RL conducted focus groups interviews, contributed to the transcription of several interviews, and wrote the first draft of the manuscript. All authors performed qualitative data analysis and contributed to manuscript revision, read, and approved the final version.

## Funding

This work was supported by FCT—Fundação para a Ciência e a Tecnologia, I.P., under grant (number PTDC/PSI-GER/28530/2017). This work received national funding from FCT—Fundação para a Ciência e a Tecnologia, I.P., through the Research Center for Psychological Science of the Faculty of Psychology, University of Lisbon (UIDB/04527/2020; UIDP/04527/2020).

## Conflict of Interest

The authors declare that the research was conducted in the absence of any commercial or financial relationships that could be construed as a potential conflict of interest.

## Publisher’s Note

All claims expressed in this article are solely those of the authors and do not necessarily represent those of their affiliated organizations, or those of the publisher, the editors and the reviewers. Any product that may be evaluated in this article, or claim that may be made by its manufacturer, is not guaranteed or endorsed by the publisher.

## Footnotes

^1^Diário de Notícias (2018). Ensino superior. https://www.dn.pt/lusa/ensino-superior-lisboa-com-30-de-alunos-deslocados-mas-so-92-tem-residencias-universitarias-9820443.html (Accessed January 15, 2022).

^2^Robert Kelchen (2018). College students’ living arrangements. https://robertkelchen.com/2018/05/28/a-look-at-college-students-living-arrangements/#_ftn2 (Accessed January 15, 2022).
